# Valuing Mortality Risk Reductions and Health Improvements: A Life Expectancy Framework to Harmonize Policy Traditions

**DOI:** 10.1111/risa.70221

**Published:** 2026-03-14

**Authors:** Jytte Seested Nielsen, Susan Chilton, Rebecca McDonald

**Affiliations:** ^1^ Newcastle University Business School (Economics) Frederick Douglass Centre Newcastle Helix, 2 Science Square Newcastle upon Tyne UK; ^2^ Department of Public Health Danish Centre for Health Economics University of Southern Denmark Odense Denmark; ^3^ Department of Economics University of Birmingham University House, Edgbaston Birmingham UK

**Keywords:** life expectancy, value per statistical life, value per statistical life year, willingness‐to‐pay for a quality‐adjusted life year

## Abstract

Many public policies directly affect an individual's mortality risk. The value of longevity gains has traditionally been monetized through three different concepts: value per statistical life (VSL), value per statistical life year (VSLY), and willingness‐to‐pay for a quality‐adjusted life year (WTP–QALY). By adopting gains in life expectancy as the unifying metric and combining it with preference‐based information on health and longevity, we set out an approach that would generate three measures that are conceptually linked at the individual level. In particular, explicitly integrating preferences over health impacts would allow for the WTP–QALY to be estimated alongside the VSL and VSLY. We argue that our approach has advantages over the direct and modeling methods developed in the literature to date. In addition, we clarify how using the proposed framework could deliver values allowing for consistent decision‐making across policy domains and government departments in ex ante regulatory assessment while simultaneously honoring the different empirical estimation practices across government departments.

## 1 Introduction

This perspective builds upon earlier theoretical work on the value of changes in life expectancy, such as Rosen (1988), Johannesson et al. (1997), Hammitt (2013), and Jones‐Lee et al. (2015). The novelty of this article is to use gains in life expectancy as a unifying metric to set out how the three concepts, value per statistical life (VSL), value per statistical life year (VSLY), and the willingness‐to‐pay for a quality‐adjusted life year (WTP–QALY), can be fully theoretically compatible with each other at the individual level. We also provide an accompanying empirical strategy whose implementation would allow estimates of all three measures to be generated.

The starting point for our framework is the valuation of avoiding a particular health state for a fixed duration. Integrating the valuation of health impacts directly into the framework allows for a derivation of the WTP–QALY using existing methods. VSLY (VSL) can similarly be elicited by linking the valuation of avoiding the health state to the gain in life expectancy (reduction in mortality risk) that the individual perceives to be equivalently beneficial. The proposed approach has a number of advantages, not least that it could deliver values that allow for consistent decision‐making across government departments while continuing to allow policymakers the flexibility to adopt whichever measure best fits their departmental policy traditions.

The United Kingdom, for example, has a long tradition of using the concept of VSL (or, equivalently, the value of a prevented fatality [VPF]) as the ex ante preference‐based measure for monetizing the value of mortality risk reductions in government decision‐making (“Green Book,” HM Treasury 2022).^1^ VSL was initially developed for the appraisal of traffic safety policies. However, government guidance (HM Treasury 2022) also recommends the use of the VSLY, which has predominantly been used in the environmental domain, and the WTP–QALY typically, used in the health domain, where one QALY is equivalent to 1 year of life in full health. Having three different concepts to monetize the value of mortality impacts may introduce incompatibilities and inconsistencies, especially if the monetary values are generated using empirical strategies with very different underlying theoretical foundations (see, e.g., Hammitt (2002) and Gyrd‐Hansen (2005) for a discussion). We provide a potential solution to this problem.

Section 2 outlines how mortality impacts can be transparently measured. In Section 3, we provide insights into the monetary valuation of risk reductions and life expectancy gains, including a unifying framework linking the three valuation concepts. In Section 4, we discuss the implications of our work and draw conclusions.

## 2 Quantifying Mortality Impacts

**FIGURE 1:**
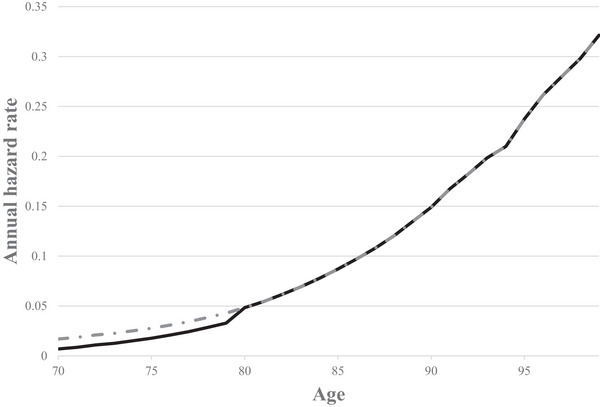
Illustrative annual hazard rates by age. The dotted curve displays the baseline hazard rates from the United Kingdom for a 70‐year‐old (National Life Tables, UK, Years 2018–2020), and the solid curve demonstrates an immediate one‐decade reduction in hazard rate.

This section outlines how mortality impacts can be quantified in a transparent way, highlighting the importance of the long‐term impacts of a given reduction in hazard rate, and describes a simple model for quantification. We set aside the issue of monetary valuation until Section 3. Here, we begin with the basic building blocks for quantifying the impact of a reduction in fatality risks. An individual's “hazard rate,” *p_t_*, for year *t* is the probability that the individual will die during that year, conditional on surviving to the beginning of the year. The relationship between hazard rate and age is illustrated by the dotted curve in Figure 1.

There are many ways in which the individual's hazard rates could be changed through different policies. For simplicity, in this perspective, we will adopt a one‐period mortality risk reduction setting, which is commonly used in safety policymaking, changing the hazard rate only in the current period. This is equivalent to a “blip” in the survival curve as introduced in Johannesson et al. (1997). The changed hazard rates are illustrated by the solid curve in Figure 1, with the hazard rates reduced for one decade.^2^

**FIGURE 2:**
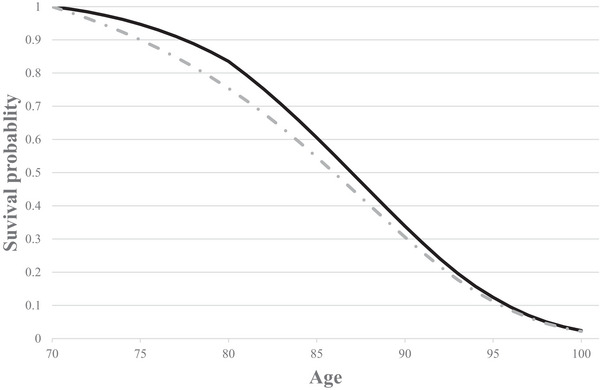
Illustrative survival curves. The dotted curve is the baseline survival curve from the United Kingdom for a 70‐year‐old (National Life Tables, UK, Years 2018–2020), and the solid curve demonstrates the survival curve with an immediate one‐decade reduction in hazard rate.

As described in Hammitt (2007) and Hammitt et al. (2020), the survival curve plots the probability that an individual is still alive as a function of her age. A baseline survival curve is illustrated in Figure 2 (dotted curve), using the same data as in Figure 1. Life expectancy at any age is the area under the survival curve that begins at that age, and reducing the hazard rate in one or more years will shift the survival curve outward by generating a gain in life expectancy. Thus, the change in the hazard function in Figure 1 can also be illustrated in Figure 2 (solid curve). The change in life expectancy is the area between the old and the new survival curves. As such, a change in life expectancy is a unifying way of representing changes in mortality risks that can capture a change in the hazard rate in one period only or in multiple periods; see also Rabl (2006) for a discussion. Although the hazard rate reduction (illustrated in Figure 1) only impacted the first period (decade), the survival curves (in Figure 2) are impacted for the rest of the individual's life.

Quantifying the policy output in terms of life expectancy gains allows the analyst to encapsulate all the potential ways that a change in hazard rates could impact an individual throughout their lifetime. This approach means that any impacts on survival probability from a change in hazard rate in any subsequent period or periods are included in the measurement. This is because, from an ex ante perspective, all mortality risk‐reducing policies would generate two effects: a “safety effect” (the direct change in hazard rate, which lasts only for the period of the risk reduction) as well as a “survivor effect” (the indirect effect of increasing the remaining survival probabilities after the “blip,” even though the hazard rate is unchanged). The intuition is that a risk reduction in an early period of life not only improves the chance of surviving in that period (the “safety effect”) but, consequently, also improves the chance of being alive in future periods (the “survivor effect”). Thus, the two effects are intimately linked, simply because any “survivor effect” is an inescapable consequence of the “safety effect.” The importance of the survivor effect is further emphasized in Section 2.1, and both effects are included in the two‐period model in Section 2.2 and discussed in Nielsen et al. (2010).

### 2.1 The Importance of the “Survivor Effect”

The “survivor effect” is important in two different but interconnected ways: (1) A large proportion of the total effect on the survival curve of a “blip” in hazard rate is a consequence of “survivor effect,” and (2) the “survivor effect” will constitute a larger proportion of the total effect for younger than for older individuals. Below, we introduce two thought experiments that enable us to consider the “survivor effect” more concretely.

First, consider the appraisal of two vaccine development and delivery programs: One that would protect against a second potential Covid‐19 pandemic (C2) and one that would protect against a second potential Spanish Flu (SF2) pandemic. For simplicity, we assume that each pandemic would last for one year and then be resolved. In both cases, the vaccine would generate a “safety effect” in that year only, with associated “survivor effect.” We assume that mortality from C2 mostly affects older members of the population (like Covid‐19), whereas SF2 causes high excess mortality among young adults (like the Spanish Flu; see Gagnon et al. (2013)). Due to the difference in age profile, a similarly sized one‐period reduction in the hazard rate would generate more “survivor effect” from a vaccine against SF2 than from a C2 vaccine.^3^ Measuring the expected gain in life expectancy in each case would capture this difference in overall benefit as a gain in life expectancy, capturing “safety” and “survivor effects.”

As a second “thought experiment,” see Table 1, where we present the implications of reducing the all‐mortality hazard rate in one year only for a cohort of 100,000 70‐year‐olds and a cohort of 100,000 30‐year‐olds (using UK statistics). For both cohorts, we reduce the hazard rate by 0.0004 in Year 1, which reduces the risk in Year 1 for a 30‐year‐old to 0.0002 and for a 70‐year‐old to 0.0155. We model the impact of this change in hazard rate on life expectancy, which is a combination of “safety” and “survivor effects.” The gain in life expectancy for the older group is 2.3 days, and the gain is more than 3 times as large for the younger group at 7.5 days. This difference in life expectancy gain is driven entirely by the difference in “survivor effect” between the two age cohorts.

**TABLE 1:** Gains in life expectancy associated with the reduction of the hazard rate in Year 1 by 0.0004.

	70 years old	30 years old
Baseline hazard rate in Year 1	0.0159	0.0006
Year 1 hazard rate reduction	0.0004	0.0004
Gain in life expectancy (LE)		
Total gain in LE	2.25 days	7.46 days
Gain in LE from “safety effect” only	0.15 days (7% of total effect)	0.15 days (2% of total effect)
Gain in LE from “survivor effect”	2.10 days (93% of total effect)	7.35 days (98% of total effect)

*Note*: Cohort of 100,000 in each age group, using UK statistics.

For illustrative purposes, we isolate the impact of the “safety effect” on life expectancy by estimating the life expectancy gains taking into account only the “safety effect” and setting the “survivor effect” to zero. The modeled life expectancy gain driven purely by the “safety effect” is equal to 0.15 days^4^ and is identical between the age groups. As highlighted in Table 1, the “survivor effect” accounts for most of the total effect (98% for the 30‐year‐olds and 93% for the 70‐year‐olds) and constitutes a larger proportion of total effect for younger individuals.

Up to now, we have outlined the links between hazard rates, survival curves, and gains in life expectancy. In the next section, we set out a two‐period model to enable us to transition from *quantifying* the impact of changes in mortality risk to the economic framework underpinning the monetary *valuation* of these changes (to be further addressed in Section 3).

### 2.2 Two‐Period Model for Quantifying Mortality Impacts

The two‐period model set out below provides us with a relatively simple set‐up while providing enough flexibility to convey the key messages. With time, t, measured in periods from the present (*t* = 1 denotes the coming period and *t* = 2 the period after), an individual's hazard rate, *p_t_*, is the probability that the individual will die during period *t*, conditional on surviving to the beginning of the period. The individual's probability of surviving period 1 is $\left(1-p_{1}\right)$ and the probability of surviving period 2 is $\left(1-p_{1}\right)\left(1-p_{2}\right)$ . Assuming for simplicity that if death is to occur in period *t*, then it does so at the beginning of the period, it follows that viewed from the present, the individual's remaining life expectancy, LE, is given by^5^

(1) $$\mathrm{LE}=\left(1-p_{1}\right)+\left(1-p_{1}\right)\left(1-p_{2}\right)$$

Consider a one‐off reduction δ (δε(0,p)) in the hazard rate in period 1. As emphasized by Cropper and Sussman (1990), when the conditional probability of death is altered in period 1, it affects the probability of surviving period 2. With this risk reduction, life expectancy becomes

(2) $$LE_{\mathrm{one}-\mathrm{off}}=\left(1-\left(p_{1}-\delta \right)\right)+\left(1-\left(p_{1}-\delta \right)\right)\left(1-p_{2}\right)$$

The impact on surviving period 1 is the “safety effect” and the impact on surviving period 2 the “survivor effect.” The gain in life expectancy is then the difference between Equations (1) and (2) as follows:

(3) $$\Delta \mathrm{LE}=LE_{\mathrm{one}-\mathrm{off}}-\mathrm{LE}=\delta \left(2-p_{2}\right)=\delta \frac{\mathrm{LE}}{1-p_{1}}$$

Hence, ΔLE equals δ times life expectancy conditional on surviving period 1. With $p_{1}$ small (as will typically be the case for anyone below the age of 80), a close approximation of ΔLE in Equation (3) will be δLE. The “safety effect” on the gain in life expectancy (equivalent to row 3 in Table 1) is equal to δ, and the survivor effect is, therefore, the difference between Equation (3) and δ, that is, $\delta (1-p_{2})$.^6^

This section has outlined the formal framework for quantifying a gain in life expectancy. For the purpose of ex ante policy evaluation (e.g., cost‐benefit analysis), this must be monetized using either the VSL, VSLY, or WTP–QALY. Despite their apparent differences, all three measures value outcomes that include both “safety” and “survivor” effects. Section 2 made the quantification step explicit, and Section 3 presents an approach for mapping these quantities to the three monetary measures.

## 3 Monetary Valuation

As outlined above, a mortality risk reduction can be represented as a change in life expectancy, and so it follows that for monetary valuation, estimating the WTP for a change in life expectancy would allow such a risk reduction to be valued. This WTP reflects the value of the gain in lifetime expected utility generated by the change in hazard rate. Traditionally, different approaches have been used across government departments to estimate the value of this gain, as described in Section 1. These existing estimation procedures can either involve direct estimation (using stated or revealed preferences^7^) or take a “modeling” approach, deriving VSLY or WTP–QALY from the VSL.

### 3.1 Direct and Modeling Approaches

To directly estimate the VSL, stated preference surveys have commonly been used to elicit the mean WTP for a mortality risk reduction from a sample of the general population; see, for example, Jones‐Lee et al. (1985), Krupnick et al. (2002), and Hammitt et al. (2019). A VSLY has been directly estimated as WTP for a gain in life expectancy; see Grisolía et al. (2018) and Chilton et al. (2004) for UK applications. Finally, WTP–QALY has been estimated as WTP for one full QALY gain; see, for example, Pennington et al. (2015) and Ahlert et al. (2016), and using the “chaining” procedure, see, for example, Gyrd‐Hansen (2003), Pinto‐Prades et al. (2009), Baker et al. (2010), and Robinson et al. (2013). Estimating the three concepts using these different approaches can be described as “fragmented” as they have developed from different traditions, leading to different estimates with no obvious common conceptual underpinning. See, for example, Hammitt (2002) and Gyrd‐Hansen (2005) for a discussion of some of the different underlying assumptions for WTP, QALY, and WTP–QALY.

As an alternative, the “modeling” approaches involve using the same, already established VSL to derive the VSLY and WTP–QALY by combining the VSL with some measure of (quality‐adjusted) life expectancy (see, e.g., Mason et al. (2009), Hirth et al. (2000), and Hammitt (2023)). Using this approach, a VSLY(*t*) is estimated as VSLY(*t*) = $\frac{\mathrm{VSL}(t)}{\mathrm{LE}(t)}$, where *t* is the individual's age and LE(*t*) can be discounted or undiscounted. A WTP–QALY(*t*) is estimated as WTP–QALY(*t*) = $\frac{\mathrm{VSL}(t)}{\mathrm{QALE}(t)}$ where QALE is quality‐adjusted life expectancy at age *t*, which can again be discounted or undiscounted. QALE(*t*) is derived by applying a quality weight to the probability of each potential year of life, which gives the *expected* QALYs (Hammitt 2023). We discuss the difference between QALYs and QALE(*t*) in Section 3.2.

A potential complication in this modeling approach is that VSLY and WTP–QALY may not reflect the same assumptions about individuals’ expected future health. We return to this point in Section 4. We address some of the issues above by setting out a framework that unifies the three concepts both conceptually and empirically.

### 3.2 Unifying Framework

Continuing with the one‐period mortality risk reduction setting and using the terminology from Section 2.2, the VSL is, on the individual level, defined as

(4) $$\mathrm{VSL}=\frac{\mathrm{WT}P_{\delta_{i}}}{\delta_{i}}$$

where $\mathrm{WT}P_{\delta_{i}}$ is individual i’s WTP for a one‐period mortality risk reduction of $\delta_{i}$. The standard approach in stated preference studies is for all respondents in the survey to value an identical δ.^8^

Similarly, the VSLY can be defined as

(5) $$\mathrm{VSLY}=\frac{\mathrm{WT}P_{\Delta LE_{i}}}{\Delta LE_{i}}$$

where $\mathrm{WT}P_{\Delta LE_{i}}$ is individual i’s WTP for a gain in LE of $\Delta LE_{i}$. As explained in Section 2.2, for any individual, ΔLE is approximated by δ LE, and we use the latter for the rest of the article.

Similarly, WTP–QALY can be defined as

(6) $$\mathrm{WTP}-\mathrm{QALY}=\frac{\mathrm{WT}P_{\Delta \mathrm{QAL}Y_{i}\;}}{\;\Delta \mathrm{QAL}Y_{i}}$$

where $\mathrm{WT}P_{\Delta \mathrm{QAL}Y_{i}}$ is individual i’s WTP for a gain in QALYs of $\Delta \mathrm{QAL}Y_{i}$.

Note that a change in QALYs is not the same as (a change in) QALE. QALYs are calculated by multiplying the time spent in a particular health state by the corresponding quality weight. QALE measures the total number of quality‐adjusted years a person can *expect* to live from a given time point. QALE effectively extends the concept of QALYs by adjusting the survival function with health‐related quality of life in each time period; see also Brown et al. (2013).

In our framework, we constrain the three WTPs ($\mathrm{WT}P_{\delta_{i}}$, $\mathrm{WT}P_{\delta LE_{i}}$, and $\mathrm{WT}P_{\Delta \mathrm{QAL}Y_{i}}$) in Equations (4)–(6) to reflect the same underlying change in utility delivered in different ways: by a one‐period change in mortality risk (VSL); a gain in (discounted)^9^ life expectancy (VSLY); or a QALY gain (WTP–QALY). This means that in practice, we identify a one‐period mortality risk reduction of $\delta_{i}$, a gain in LE of $\delta_{i}LE_{i}$, and a gain in QALY of $\Delta \mathrm{QAL}Y_{i}$ between which an individual is indifferent. To do this, we begin with a nonfatal injury or illness, then find the value of that injury or illness, and finally find the one‐period mortality risk reduction, the gain in LE, and the gain in QALYs that the individual judges to be equally valuable as the avoidance of that nonfatal injury or illness.^10^ In the following abridged version of the framework presented in Chilton et al. (2020), based on the chained‐elicitation method, we explain how this works in practice.

First, we will outline how our framework can estimate VSL and VSLY. This will be followed by an extension to WTP–QALY in Section 3.2.2. In Section 3.2.3, we will discuss the relationship between WTP–QALY and VSLY.

#### 3.2.1 Estimating VSL and VSLY

Fundamentally, three main components are needed for the framework (we return to Component 3 in Section 3.2.2).

**Component 1**: Elicitation of WTP (using stated preference techniques) for a quick and complete cure for a nonfatal injury or illness (lasting for 1 year followed by a permanent return to normal health).

**Component 2**: Elicitation of the gain in life expectancy in normal health $\delta_{i,N}LE_{i,N}$ 11 that is as good as a quick and complete cure for the injury or illness in Component 1.

First, we set out how Component 2 $\delta_{i,N}LE_{i,N}$ could be elicited empirically in this case using a modified standard gamble (SG) (see Carthy et al. (1999)). Denote the utility of spending 1 year in *normal (N) health* by *H_N_* and the utility of spending 1 year with the injury/illness by *H*_1,_ *_N_*, where the reference state is normal health. $\delta_{i,N}$ can be elicited as the maximum risk of treatment failure (resulting in death) that an individual would be prepared to accept in a treatment, which, if successful, would result in an immediate and complete cure for the injury or illness and returning to normal health for the rest of their life. In addition, for $\delta_{i,N}LE_{i,N}$ to be derived, LE*_i_*_,_ *_N_* is required, which can be estimated (objectively) from statistical life tables or alternatively by eliciting the individual's subjective life expectancy (see, e.g., Hamermesh (1985); Rappange et al. (2017)). Hence, from the modified SG, we can get $\;\delta_{i,N}LE_{i,N\;}$, which is the life expectancy gain in normal health yielding the same gain in lifetime expected utility as a quick and complete cure for the injury/illness that would have lasted for 1 year. To express the trade‐off in the modified SG formally, let LE*_i,N_* represent the individual's life expectancy in normal health. Under the assumptions of expected utility theory (EUT), assuming utility is linear in duration and setting the utility of death at zero, it follows that^12^

(7) $$H_{1,N}+\left(LE_{i,N}-1\right)H_{N}=\left(1-\delta_{i,N}\;\right)H_{N}\;LE_{i,N}$$

from which follows that

(8) $$\delta_{i,N}\;LE_{i,N}=1-H_{1,N}/H_{N}$$

From this, VSL and VSLY on the individual level can be estimated using Equations (4) and (5).

#### 3.2.2 Elicitation of WTP–QALY

In the following, we will explain how the WTP–QALY can be derived on the individual level (Equation 6). For this, we need a third component.

**Component 3**: Elicitation of the $\Delta \mathrm{QAL}Y_{i,F}$ that is as good as a quick and complete cure for the injury or illness in Component 1.

Component 3 could be derived empirically using a time trade‐off (TTO).^13^ Denote the utility of spending 1 year in *full (F) health* by *H_F_* and the utility of spending 1 year with the injury/illness by *H*_1,_ *_F_*, where the reference state is full health. Suppose that in response to a TTO question, an individual indicates that spending T years in *full health* would be equally as desirable as spending 10 years with the particular illness/injury.^14^ In this example, $10H_{1,F}=TH_{F}$ so that the QALYs associated with 1 year of suffering the illness/injury are $\frac{H_{1,F}}{H_{F}}=\frac{T}{10}$ and hence

(9) $$\Delta \mathrm{QAL}Y_{i,F}=1-H_{1,F}/H_{F}$$

From here, WTP–QALY on the individual level can be estimated using Equation (6).

#### 3.2.3 Relationship Between VSLY and WTP–QALY

In this section, we set out a proposition that outlines the relationship between VSLY and WTP–QALY. First, though, it is useful to consider the underlying requirements that must be fulfilled for the VSLY and WTP–QALY to be equal.

Comparing the modified SG approach to estimating a VSLY (described in Section 3.2.1) and the TTO approach to estimating a WTP–QALY (described in Section 3.2.2), it is clear that if (in the special case) the reference state “normal health” used in the modified SG is equal to “full health” (used in the TTO), we have that *H_N_* = *H_F_* = 1 and *H*_1,_ *_N_* = *H*_1,_ *_F_*. Using these relationships, it follows from Equations (8) to (9) that $\left(1-H_{1,N}\right)=\left(1-H_{1,F}\right)$ leading to the equivalence $\;\Delta \mathrm{QAL}Y_{i,F}=\delta_{i,N}LE_{i,N}$.

On this basis, we set up the following proposition, proof sketch, and example:

Proposition: *Under expected utility with utility linear in duration, separability of wealth and health, and the appropriate discounting, if and only if “normal health” = “full health,” then VSLY = WTP–QALY. When the reference states differ (“normal health” < “full health”), then VSLY < WTP–QALY*.

Proof sketch: When *H_N_* ≤ *H_F_* =1, the special case of $\Delta \mathrm{QAL}Y_{i,F}=\delta_{i,N}LE_{i,N}$ extends to

(10) $$\Delta \mathrm{QAL}Y_{i,F}=\delta_{i,F}\;LE_{i,F}=\left(\delta_{i,N}\;LE_{i,N}\right)H_{N}$$

where $\delta_{i,F}\;LE_{i,F}$ is a gain in life expectancy with reference level “full health.” Given *H_N_* ≤1, it follows that $\delta_{i,F}\;LE_{i,F}\leq \delta_{i,N}\;LE_{i,N}$ which leads to

(11) $$\Delta \mathrm{QAL}Y_{i,F}\leq \delta_{i,N}LE_{i,N}$$

As WTP in the framework is independent of whether the TTO is used to elicit $\Delta \mathrm{QAL}Y_{i,F}$ or the modified SG is used to elicit $\delta_{i,N}LE_{i,N}$, from Equations (5) to (6) and (11) follows the general relationship that VSLY ≤ WTP–QALY with equality between VSLY and WTP–QALY (in the special case) if and only if *H_N_* = *H_F_*.

To illustrate,^15^ set *H_N_* = 0.92, $\delta_{i,N}$ = 0.012, and $LE_{i,N}$ = 15.

$$\delta_{i,N}\;LE_{i,N}=0.18$$

$$\;\Delta \mathrm{QAL}Y_{i,F}=\left(\delta_{i,N}\;LE_{i,N}\right)H_{N}=0.1656$$

As $\delta_{i,N}LE_{i,N}>\Delta \mathrm{QAL}Y_{i,F}$ and with WTP constant and *H_N_* < 1, it follows that VSLY < WTP–QALY.

ΔQALY*_i,F_*_,_ and $\delta_{i,N}LE_{i,N}$ will vary to the extent that the perception of the utility in the reference state “normal health” (*H_N_*) differs from that of “full health” (*H_F_*). The extent to which *H_F_* differs from *H_N_* is an open empirical question.

In summary, we have illustrated for a given gain in health and duration how the chained‐elicitation method can unite VSL, VSLY, and WTP–QALY for the individual. It does this by adopting gains in life expectancy as the unifying metric and through the explicit integration of the valuation of a health state, which allows for the WTP–QALY^16^ to be incorporated within the framework. In addition, the framework controls for the individual's future health in the elicitation by making expected future health explicit in the empirical survey scenario.

## 4 Discussion

We set out an approach, which is based on the chained‐elicitation method, that would generate three measures to value changes in safety and health that are conceptually linked at the individual level.^17^ To do so, we adopt gains in life expectancy as the unifying metric and combine them with information about preferences about safety and health. In particular, the advantage of explicitly integrating health impacts within this framework is that it would allow for the WTP–QALY to be estimated alongside the VSL and VSLY. In addition, implementing the proposed valuation framework builds on the foundation for measuring mortality impacts set out in Section 2, describing how gains in life expectancy can explicitly take into account “survivor effect” as well as the “safety effect” and would be fully in accordance with an ex ante perspective for policy evaluation. Under our valuation framework, we make it transparent how all three concepts (VSL, VSLY, and WTP–QALY) are related to each other on an individual level because the estimation of all three is based on gains in life expectancy. This has advantages over previous approaches, for example, direct elicitation approaches that separately estimate each concept and modeling approaches. As discussed in Section 3.1, using direct approaches to separately estimate each concept could be problematic, because this fragmented approach generates values that lack any obvious common conceptual underpinning. The benefits of the proposed framework are further enhanced by its reliance on only one stated preference data set, which is also in contrast to the “modeling” approaches. In the “modeling” approaches, as quality‐adjusted weights are not integrated in standard VSL surveys, they have to be adopted from secondary sources, and the preferences, reflected in a WTP–QALY, will therefore be based on two different population samples.^18^

A further concern for policymaking is that there will be systematic differences between WTP–QALY and VSLY that are driven by the different assumptions about future health that are made between empirical settings. In the “modeling” approaches, the expectations about future health are not observable to the researcher in the VSL setting, and the assumptions made about future health differ between the modeling approach that delivers the VSLY and the approach that delivers the WTP–QALY. By contrast, in our proposed framework, we explicitly set the expectation of normal or full health in the modified SG and the TTO. A further requirement in our framework is a derivation of life expectancy for the individual. For policymaking, objective measures can be used as an approximation. If unbiased estimates of subjective life expectancy could be elicited, these could be used as an alternative.^19^

In the unifying framework, we demonstrate the compatibility of the three concepts at the individual level. The advantage of our approach is that—should the policymaker use these measures interchangeably across policy domains—they can be confident that they are generated from the same underlying preferences over health and longevity. If values are elicited for a nationally representative sample of individuals, then policymakers could reasonably assume these preferences mapped to the aggregate values that should underpin regulation. Although we argue that this is an improvement on existing approaches, in practice, the values must be aggregated across individuals to make them applicable across policy settings. This aggregation and application bring in additional challenges—common to all approaches, including our own—for example, when policy beneficiaries are different ages and hence have different remaining life expectancies. Hammitt (2023, 2) summarizes the approach most commonly adopted in policymaking, that is, it is “common practice to value a life‐saving policy by assuming that one of [the VSL, VSLY, WTP–QALY] is constant (independent of age and other individual attributes),” and discusses the inconsistencies that arise as a result.

So, although these aggregation issues are clearly relevant, they are orthogonal to the link between the three concepts at the level of the individual and the empirical strategy we have described. It would be inappropriate to use our framework to present any policy recommendation with respect to this issue since it does not address consistency at this level. Instead, eliciting the values within our framework resolves the incompatibilities at the individual level that could arise when policymakers use values from different sources, methods, and data. This allows for policy traditions to be harmonized with respect to the valuation of changes in safety and health.

Although our findings present a step forward in understanding how to bring the three policy traditions into a unifying framework, there is much yet to learn. Future research could usefully extend the model framework using, for example, nonlinear duration utility or rank‐dependent models. Retaining the one‐period risk reduction approach that underpins current UK values is pragmatic and allows us to demonstrate the link between the three concepts with the one‐period WTP–QALY and VSL as cornerstones. An obvious next step is to explore the valuation of multi‐period risk reduction, investigating how the VSLY varies with the way that a given gain in life expectancy is generated. Methods to elicit relative preferences over a given change in life expectancy generated by different perturbations to the survival function have already been developed, in which participants make pairwise choices between different ways of generating the same gain in life expectancy (Nielsen et al. 2010; Hammitt and Tunçel 2015).^20^ Members of the general population were found to have preferences over different ways of generating a given gain in life expectancy, indicating VSLYs should be sensitive to how the life expectancy gain is generated. In principle, relative preferences between different ways of generating the same gain in life expectancy could be “pegged” to a VSLY from the one‐period risk reduction framework presented here to derive VSLYs for different multi‐period risk reductions. If gains in life expectancy became the underlying basis for quantification of mortality impacts, further theoretical and empirical refinements would almost certainly follow, with the potential to further improve the consistency of government policymaking and the accurate representation of people's preferences in resource allocation.

## Conflicts of Interest

The authors declare no conflicts of interest.

## Data Availability

No data were collected for the preparation of this perspective.

## Endnotes

1 The VSL is based in expected utility and set out in Drèze (1962) and Jones‐Lee (1974).

2 The illustration is based on hazard rates from the United Kingdom for a 70‐year‐old. The insights drawn from Figure 1 would apply to any hazard rate reduction at any age. However, the pattern would be imperceptible for younger individuals in the survival curve illustration (Figure 2) due to their very low hazard rates. Similarly, for shorter risk reductions (e.g., 1 year), the survival curve illustration would be imperceptible.

3 Any hazard rate reduction will generate “survivor effects” that benefit all future periods of life. As a result, the earlier the hazard rate reduction, the more future periods exist to benefit from the survivor effect, and hence, the greater the survivor effect will be, ceteris paribus.

4 The gain in life expectancy is calculated as 0.0004 for 1 year × 365 days/year.

5 Each year's survival probability is multiplied by 1, representing 1 period.

6 This means that the “survivor effect” is decreasing when the period two hazard rate increases and, hence, decreasing when period two survival probability decreases. As hazard rates increase with age, the “survivor effect” of a given risk reduction is smaller for older recipients, as was also illustrated in Table 1.

7 We focus on the stated preference literature as it is predominant in the United Kingdom.

8 For simplicity, Equations (4)–(6) are presented on the individual level. In cost‐benefit analysis values are summed across the relevant population, and hence, Equations (4)–(6) would be estimated on the aggregate level in line with the UK definition of VSL, as discussed in Jones‐Lee et al. (1985).

9 Here and hereafter, life expectancy should be subject to appropriate discounting using the appropriate rate of personal time preference. In what follows, we refer to life expectancy with the understanding that any discounting is incorporated. See also Chilton et al. (2020) and Jones‐Lee et al. (2015) for a discussion.

10 The unifying framework would hold for any point on the individual's marginal WTP for gains in life expectancy curve; see Chilton et al. (2020).

11 According to Equation (3), ΔLE is approximated by δ LE_._ The exact equality, in Equation (3), would apply if the empirical approach began from the next period. It would be possible to extend the framework to use this more precise equality, but we present the framework to include the possibility of fatality in the current period (which accords with the standard VSL model).

12 In what follows, we assume separability of wealth and health as well as additivity over time. We refer the reader to Bleichrodt and Quiggin (1999) and Herrera‐Araujo et al. (2020) for alternative assumptions.

13
*H*_1_ can alternatively be elicited using an SG where the injury/illness is chronic (rather than temporary as in the modified SG); see Drummond et al. (2005) for an example. With a non‐modified SG, $H_{1,F}/H_{F}$ = (1 − $\delta_{i,F})$, refer to Chilton et al. (2020) for details.

14 In the TTO exercise, the 10‐year period is conventionally followed by death; see Lugnér et al. (2020).

15 The value of $\delta_{i,N}$ is taken from Carthy et al. (1999), Table 1, and $LE_{i,N}$ = 15 is under the assumption that average remaining life expectancy is 40 years, which, applying a discount rate of 6%, converts to 15 discounted life years.

16 There is evidence that WTP–QALY is not independent of the size of the health gain individuals are asked to value, whether the health gain is constant or uncertain, and whether the gain represents improvements in health quality or changes in duration of the symptoms (see, e.g., Pinto‐Prades et al. (2009) and Nielsen et al. (2021)). For this perspective, we sidestep these issues as we analyze consistency within an individual (between VSLY, VSL, and WTP–QALY) for a given gain in health and duration.

17 Note that when estimated empirically, the chained method is based on answers to multiple questions, and therefore, the standard errors need to be carefully estimated across the different components.

18 For example, Mason et al. (2009) used the weights from Kind et al. (1999), and these were assumed to be constant and based on an average across the population; and Hammitt (2023) used empirically derived age‐dependent health‐related quality of life from Hanmer and Kaplan (2016).

19 We refer to Viscusi and Hakes (2003) and Rappange et al. (2017) for a discussion of some of the inconsistencies found when estimating subjective life expectancy.

20 More recently, this work has been extended to also elicit WTP directly for different distributions of risk reduction that produce different gains in life expectancy (Hammitt and Tuncel 2023).
